# Post-conversion targeted capture of modified cytosines in mammalian and plant genomes

**DOI:** 10.1093/nar/gkv244

**Published:** 2015-03-26

**Authors:** Qing Li, Masako Suzuki, Jennifer Wendt, Nicole Patterson, Steven R. Eichten, Peter J. Hermanson, Dawn Green, Jeffrey Jeddeloh, Todd Richmond, Heidi Rosenbaum, Daniel Burgess, Nathan M. Springer, John M. Greally

**Affiliations:** 1 Department of Plant Biology, University of Minnesota, 1445 Gortner Ave, Saint Paul, MN 55108, USA; 2 Center for Epigenomics and Division of Computational Genetics, Department of Genetics, Albert Einstein College of Medicine, 1301 Morris Park Avenue, Bronx, NY 10461, USA; 3 Roche-NimbleGen, 500 South Rosa Road, Madison, WI 53711, USA

## Abstract

We present a capture-based approach for bisulfite-converted DNA that allows interrogation of pre-defined genomic locations, allowing quantitative and qualitative assessments of 5-methylcytosine (5mC) and 5-hydroxymethylcytosine (5hmC) at CG dinucleotides and in non-CG contexts (CHG, CHH) in mammalian and plant genomes. We show the technique works robustly and reproducibly using as little as 500 ng of starting DNA, with results correlating well with whole genome bisulfite sequencing data, and demonstrate that human DNA can be tested in samples contaminated with microbial DNA. This targeting approach will allow cell type-specific designs to maximize the value of 5mC and 5hmC sequencing.

## INTRODUCTION

The study of DNA methylation (creating 5-methylcytosine, 5mC) has revealed it to have a number of interesting properties. It is a heritable epigenetic event in the genome, capable of replicating itself to daughter cells through recognition involving UHRF1, which recruits DNMT1 to hemi-methylated DNA to restore methylation on both strands following replication (1). It has complex relationships with gene expression, with contextual dependencies associated with promoter, CpG island or gene body location and the transcriptional status of the locus (2). DNA methylation appears to be targeted to transcribed sequences and to euchromatin (3), but is also targeted to pericentromeric heterochromatic satellite DNA sequences (4). The relationship of DNA methylation with gene activity is therefore complex and has to be interpreted within its specific genomic context.

As well as 5mC occurring in the context of a CG dinucleotide, 5mC can also be found in non-CG contexts. This can occur in mammalian pluripotent cells (5), mouse brain (6) and human brain (7,8), probably targeted by DNMT3A (8) but is a common occurrence in plant genomes (9,10), where there are enzymes whose specific functions are to direct CHG and CHH methylation (11). 5-methylcytosine is oxidized by the TET family of enzymes to 5-hydroxymethylcytosine (5hmC), which is found in higher amounts in certain cell types of mammals and appears to be produced as part of a process to remove 5mC from the genome (12).

DNA methylation is part of a complex system of transcriptional regulation involving variability in the constituents and structure of chromatin, post-translational modifications of components of chromatin, the effects of non-coding RNAs, and possibly less appreciated contributors like non-canonical nucleic acid structures (13). It has proven possible to test DNA methylation genome-wide quantitatively at nucleotide resolution, allowing insights into its distribution in normal cells and its dysregulation in disease (14,15).

Human disease studies testing for pathogenic epigenetic dysregulatory mechanisms have focused on DNA methylation analysis because in part of the relative maturity and strengths of the assays for its measurement throughout the genome. What is apparent from these human disease studies is that the degree of change of DNA methylation associated with a phenotype or disease can be very limited (16). This being the case, assays need a wide dynamic range of measurement capability, which was traditionally accomplished using various microarray approaches (17–19), with a move more recently to the adoption of assays based on the massively parallel sequencing of bisulfite-converted DNA (14–15,20). For sequencing-based assays to allow discrimination of limited changes in DNA methylation, reasonably deep average coverage has to be achieved in substantial numbers of samples, which combine to create a financial resource challenge when whole genome analysis is deemed necessary.

To circumvent this problem, various approaches have been developed to ‘survey’ the genome, testing only those loci where prior knowledge suggests their informativeness in terms of DNA methylation changes to have functional consequences, usually in terms of gene transcription. The common survey approaches include microarrays (Illumina HumanMethylation450K) (19), reduced representation bisulfite sequencing (RRBS, sequencing of bisulfite-converted small MspI fragments) (20) and restriction enzyme-based approaches exemplified by HELP-tagging (21). These survey assays make genome-wide surveys affordable and offer sufficient resolution to identify differential DNA methylation of only modest degrees.

Testing hydroxymethylation of DNA is even more challenging. It cannot be discriminated from 5mC in regular bisulfite mutagenesis assays, as neither 5mC nor 5hmC converts to uracil (22). Its presence also prevents digestion by methylation-sensitive restriction enzymes, so approaches have been developed that selectively protect the 5hmC from restriction enzyme digestion (23) or TET-mediated oxidation (24), or cause the 5hmC selectively to oxidize to 5-formylcytosine (5fC) using potassium perruthenate (KRuO_4_ (25)), followed in each case by bisulfite mutagenesis to allow the discrimination of 5hmC from 5mC. The problem with sequencing-based approaches for 5hmC is the low proportion and therefore allelic frequency of 5hmC within the population of molecules (25), requiring even more substantial read depth than is necessary for regular bisulfite sequencing, a further resource challenge.

The most informative sites for studying DNA methylation in the genome are likely to be distal *cis*-regulatory elements rather than gene promoters, with enhancers in particular where DNA methylation changes are most obviously causally correlated with transcriptional changes in human diseases (26–31) and normal cells (32). However, a drawback to current genomic survey approaches for 5mC is that they cannot take advantage of new information about the *cis*-regulatory landscape in different cell and tissue types resulting from the ENCODE program, the Roadmap Epigenomics Program and other initiatives. It is now possible to map, based on ChIP-seq data, the locations of candidate promoters and enhancers in a cell type of interest (33). Ideally, the sequencing-based approaches quantifying 5mC and 5hmC would be targeted to different sets of pre-defined *cis*-regulatory sites in different cell types, allowing the maximum value to be gained from the sequencing performed.

Recognizing the value of targeted approaches, there have been some novel assays developed that select pre-defined loci for 5mC assays. Some are based on multiplex polymerase chain reaction (PCR) (34), others on the use of padlock probes (35), while others have used variations on affinity capture of DNA followed by sequencing (36,37), the approach employed successfully for exome-sequencing, allowing a substantial proportion of the genome to be targeted in a single assay. When capturing DNA with bisulfite sequencing as the downstream goal, two choices present themselves—to capture the native DNA and then convert this material with sodium bisulfite (capture-then-convert) or to convert the DNA using bisulfite mutagenesis and then capture the resulting material (convert-then-capture). Both have been attempted previously. The first such study in 2009 used the convert-then-capture approach, targeting 324 CpG islands and generating data on >25 000 CG dinucleotides (36). The second study used the capture-then-convert alternative approach, testing 21 408 CpG islands and ∼1 million CG dinucleotides (37). A commercial system for capture-based bisulfite sequencing from Agilent Technologies (SureSelect Methyl-Seq) is based on the latter, capture-then-convert approach.

Each of these approaches has a different associated theoretical problem. In a capture-then-convert assay, a large amount of DNA is needed at the outset yielding a limited number of molecules following capture, which are then at risk of extensive degradation by the harsh effects of bisulfite treatment (38). This leads to the theoretical possibility of generating low-complexity libraries, which would manifest as having large proportions of PCR duplicates in the sequencing output and reduced information content per unit of sequence data generated. The convert-then-capture approach has a different theoretical problem, the need to ensure that the capture reagents are capable of binding to all of the many possible alleles generated by bisulfite mutagenesis. The number of possible alleles for each strand of DNA is 2^n^, where n is the number of potentially methylated cytosines in the fragment.

Here we describe a new convert-then-capture system that performs exceptionally well in generating sequences representing the full complexity of the allelic variants resulting from bisulfite conversion. We show that the assay is accurate, detecting 5mC in non-CG contexts, allowing single nucleotide polymorphisms (SNPs) to be detected, and that it can be used with relatively low amounts of input DNA and can also be applied to targeted 5hmC sequencing, with results generated from both animal and plant species. The platform is ideally suited for targeted studies of DNA methylation based on empirical annotation of *cis*-regulatory elements in a cell type of interest, providing the most powerful epigenomic genomic survey approach to date.

## MATERIALS AND METHODS

### Samples used

#### HCT116 cell line

We purchased DNA from the HCT116 cell line with a double knock out (DKO) of the *DNMT1* and *DNMT3A* genes (Zymo Research).

#### IMR90 fibroblasts

IMR90 human primary fibroblasts (ATCC CCL-186) were cultured in ATCC-formulated Eagle's Minimum Essential Medium supplemented with 10% FCS following the recommended ATCC culture protocol (http://www.atcc.org/products/all/CCL-186.aspx#culturemethod).

#### Lymphoblastoid and Burkitt's lymphoma cell lines

The NA12762 DNA sample was purchased from the Coriell Institute for Medical Research. This sample is derived from the GM12762 lymphoblastoid cell line, in turn derived from a Caucasian male who is part of the CEPH collection of pedigrees. The NA04671 DNA sample, also purchased from the Coriell Institute, is derived from the GM04671 Burkitt lymphoma cell line which is part of the NIGMS Human Genetic Cell Repository, the original tumor occurring in a Yoruban 11-year-old male.

#### Buccal epithelial samples

All patient recruitment and sample collection was performed with the appropriate human subjects protocol approval from the Institutional Review Board at the Albert Einstein College of Medicine. We have described the collection of these samples previously (39). We collected buccal epithelium using exfoliative brushing. The brushes were stored in 15 ml Falcon tubes containing 4 ml of ThinPrep CytoLyt Solution (Hologic, Inc.) immediately after the swabbing. The buccal epithelial cells were separated from the brush by shaking, and then spun down to remove the preservative solution. The pellets were stored at −80°C. DNA from the buccal epithelial samples was extracted with a modification of the protocol for the Qiagen Gentra Puregene Buccal Cell kit (QIAGEN) (39).

#### Maize samples

Maize inbred lines B73 and Mo17 as well as the F1 hybrid B73xMo17 were grown in using standard greenhouse conditions. The third leaf was harvested and used for DNA extraction using the standard CTAB method.

#### Mouse embryonic stem cells

The E14.Tg2a embryonic stem (ES) cell line was cultured in Dulbecco's Modified Eagle Media (DMEM) (Knockout DMEM, Life Technologies), containing 15% FBS (ES cell-qualified, Life Technologies), 1000 U/ml leukemia inhibitory factor (Chemicon), 0.1 mM 2-mercaptoethanol (Life Technologies) on a gelatin-coated support in the absence of feeder cells (40). To harvest the ES cells, they were dissociated with trypsin-EDTA (0.05%), and then collected by centrifugation. Genomic DNA was isolated from the cell pellet using proteinase K digestion, phenol-chloroform extraction, dialysis against 0.2x SSC, concentrating the sample by surrounding the dialysis bag with polyethylene glycol (MW 20 000) to reduce water content by osmosis. The quality of the DNA was checked by gel electrophoresis and the concentration measured using Qubit fluorometric quantitation (Life Technologies).

### Construction of libraries

We fragmented 1 μg of input genomic DNA and 5.8 μl of bisulfite-conversion control (165 pg) in a total volume of 50 μl using a Covaris E210 series shearing instrument to an average size range of 180–220 bp. Libraries were constructed using the KAPA HTP Library Preparation Kit Illumina (Roche NimbleGen), SeqCap Adapter Kit A and B (Roche NimbleGen) and SeqCap EZ Pure Capture Bead Kit (Roche NimbleGen). The 50 μl of sheared gDNA was transferred to 0.2 ml PCR strip tubes and 20 μl of End Repair Enzyme mix (8 μl of H_2_O, 7 μl of 10x KAPA End Repair buffer and 5 μl of KAPA End Repair Enzyme) were added to each tube of genomic DNA and mixed by pipetting up and down. The End Repair reactions were incubated at 20°C for 30 min. After incubation, 120 μl of room temperature SeqCap EZ Purification Beads were added to the End Repair DNA and mixed by pipetting up and down. The DNA/bead mixture was incubated for 15 min at room temperature to allow the DNA to bind to the beads. The sample tubes were then placed on a DynaMag-96 Side Magnet (Life Technologies) and the solution was allowed to clear. Without disturbing the pellet, the supernatant was removed and discarded. Beads plus bound DNA were washed twice with 200 μl of freshly prepared 80% EtOH while on the magnet. The beads plus bound DNA were allowed to dry until they were no longer glossy and all remaining ethanol had evaporated. To the dry beads with bound DNA, 50 μl of A-Tailing Enzyme mix (42 μl of H_2_O, 5 μl of KAPA A-Tailing buffer and 3 μl of KAPA A-Tailing enzyme) were added to each tube and mixed by pipetting up and down. The A-Tailing reactions were then incubated at 30°C for 30 min. Following the completion of the A-Tailing reaction, 90 μl of room temperature PEG/NaCl SPRI solution (KAPA HTP Library Preparation Kit Illumina) were added to the DNA and vortexed. The A-Tailed DNA with PEG/NaCl SPRI solution was incubated for 15 min at room temperature. The sample strip tubes were then placed on the magnet and the solution was allowed to clear. Without disturbing the pellet, the supernatant was removed and discarded. Beads plus bound DNA were washed twice with 200 μl of freshly prepared 80% EtOH while the beads were left on the magnet. The beads plus DNA were then allowed to dry until they were no longer glossy and any remaining ethanol had evaporated. To the dried beads with bound A-Tailed DNA, 45 μl of Ligation Master Mix (30 μl of H_2_O, 5 μl of 10X KAPA Ligation Buffer and 5 μl of KAPA T4 DNA Ligase) and 5 μl (10 μM) of a predetermined Index adaptor (SeqCap Adapter Kit A or B) were added. The reactions were mixed by pipetting and incubated at 20°C for 15 min. Following the Adaptor Ligation reaction, 50 μl of room temperature PEG/NaCl SPRI solution were added to the reactions and vortexed to resuspend the beads. DNA plus beads were incubated for 15 min at room temperature. The sample strip tubes were placed on the magnet and the solution was allowed to clear. Without disturbing the pellet, the supernatant was removed and discarded. Beads plus bound DNA were washed twice with 200 μl of freshly prepared 80% EtOH while the beads were left on the magnet. The beads plus DNA were allowed to dry until they were no longer glossy and any remaining ethanol had evaporated. Beads plus DNA were then resuspended in 100 μl of H_2_O. Sixty microliter of room temperature PEG/NaCl SPRI solution was added to the beads with DNA and the samples were vortexed. The DNA-bound beads were incubated for 15 min at room temperature. The sample strip tubes were placed on the magnet and allowed to clear. Without disturbing the pellet, 155 μl of supernatant was removed and put in a new 0.2 ml PCR strip tube. Then 20 μl of room temperature SeqCap EZ Purification beads were added to the tube with the supernatant. These DNA-bound beads were incubated for 15 min at room temperature. The sample strip tubes were then placed on the magnet and the solution allowed to clear. The supernatant was removed and the DNA-bound beads were washed twice with 200 μl of freshly prepared 80% EtOH while on the magnet. The beads with DNA were allowed to dry until they were no longer glossy and any remaining ethanol had evaporated and were then resuspended in 25 μl of H_2_O. The sample strip tubes were placed on the magnet and allowed to clear. Finally, 20 μl of the sample library (supernatant) was removed and transferred to a new 0.2 ml PCR tube.

### Bisulfite conversion of DNA sample libraries

One hundred thirty microliters of Lightning Conversion Reagent (EZ DNA Methylation-Lightning Kit, Zymo Research) were added to the 20 μl of the DNA Sample Library. Samples were briefly vortexed and spun down. Due to volume restrictions with our thermal cycler machine, 75 μl of the Library plus Conversion Reagent mixture were transferred to two new 0.2 ml PCR tubes. The DNA Sample Libraries were converted using the following thermal cycler program: 98°C for 8 min, 54°C for 60 min and 4°C for up to 20 h. Following the completion of the thermal cycler program, 600 μl of M-Binding Buffer were added to a Zymo Spin IC Column and placed in a collection tube. The bisulfite-converted contents of the two 0.2 ml tubes were combined and added to the sample column containing 600 μl of M-Binding Buffer. After closing the caps, the tubes were inverted 5–6 times to mix. Columns were centrifuged at full speed for 30 s. The flow-through was discarded. One hundred microliter of M-Wash Buffer (after 24 ml of 100% ethanol was added to the 6 ml of M-Wash Buffer) was added to the column and centrifuged at full speed for 30 s. Two hundred microliters of L-Desulphonation Buffer were added to the column, the tube lids were closed, and then incubated for 20 min at room temperature. After incubation, the columns were centrifuged at full speed for 30 s and the flow-though was discarded. Two washes of the columns using 200 μl of M-Wash Buffer and centrifugation at full speed for 30 s were performed. Columns were placed in a new 1.5 ml centrifuge tube for collection and 20 μl of pre-warmed PCR grade water was added to the center of the column. Columns were centrifuged at full speed for 1 min and the eluted Bisulfite-converted Sample Libraries were then amplified using Pre-Capture LM-PCR as follows.

### Pre-capture LM-PCR of bisulfite-converted sample libraries

The Bisulfite-converted Sample Library was amplified using ligation-mediated PCR (LM-PCR). The 20 μl of Bisulfite-converted Sample Library were added to a new 0.2 ml PCR tube containing the Pre-Capture LM-PCR Master Mix (25 μl of 2x KAPA HiFi Hot Start Uracil + Ready Mix, 3 μl of 5 μM Pre LM-PCR Oligo 1 and 2, and 2 μl of PCR grade water) (SeqCap Epi Accessory Kit, Roche NimbleGen). Bisulfite-converted Sample Libraries were amplified in a thermal cycler using the program: step 1: 95°C for 2 min, step 2: 98°C for 30 s, step 3: 60°C for 30 s, step 4: 72°C for 4 min, step 5: go to step 2, repeat 11 times, step 6: 72°C for 10 min and step 7: 4°C indefinitely. After the completion of the thermal cycler program, the amplified Bisulfite-converted Sample Library was transferred to a new 1.5 ml tube containing 250 μl of buffer PBI with the pH indicator added (QIAquick PCR Purification Kit, Qiagen) along with 10 μl of 3.0 M Sodium Acetate. Tubes were quickly vortexed to mix then centrifuged. The entire volume (∼300 μl) was transferred to a QIAquick Spin Column and centrifuged for 1 min at 13 000 xg. The flow-through was discarded and the column was washed with 750 μl of PE buffer (220 μl of 100% ethanol added to 55 μl of PE). The column was centrifuged for 1 min at ∼13 000 xg. The flow-through was discarded and the column centrifuged a second time to get rid of all wash buffer. The column was placed in a new 1.5 ml centrifuge collection tube and 50 μl of pre-warmed PCR grade water was added to the center of the column. The column was centrifuged for 90 s at 13 000 xg. The concentration of the amplified Bisulfite-converted Sample Library was determined using a Nanodrop Spectrophotometer (Thermo Fisher Scientific) and 1 μl of diluted sample was analyzed using a High Sensitivity DNA Chip on a 2100 Bioanalyzer instrument (Agilent Technologies).

### Hybridization

One microgram of the amplified Bisulfite-converted Sample Library was added to a new 1.5 ml centrifuge tube, with a hole pierced in the tube lid, containing 1 μl of SeqCap HE Universal Oligo (1000 μM), 1 μl of the appropriate SeqCap HE Index Oligo (1000 μM) and 10 μl of Bisulfite Capture Enhancer (SeqCap HE Oligo Kits A and B and SeqCap Epi Accessory Kit, Roche NimbleGen). With the lid closed, the tubes containing the Sample Libraries, Bisulfite Capture Enhancer and Oligos were dried down using a DNA vacuum concentrator on high heat. To the dried-down amplified Bisulfite-converted Sample Library plus Oligos and Bisulfite Capture Enhancer, 7.5 μl of 2× Hybridization Buffer and 3 μl of Hybridization Component A (SeqCap EZ Hybridization and Wash Kit, Roche NimbleGen) were added. The hole at the top of the 1.5 ml tubes was covered with lab tape and the samples were vortexed and briefly centrifuged. The samples were then heated in a 95°C heat block for 10 min. After denaturation, the samples were vortexed, allowed to return to room temperature and briefly centrifuged. The entire volume was then transferred to a 0.2 ml tube containing the SeqCap Epi Choice probe pool (Roche NimbleGen) that had been previously aliquoted in 4.5 μl amounts in 0.2 ml PCR strip tubes. The samples were mixed briefly and then incubated in a thermal cycler for 68 h at 47°C (with a heated lid).

### Binding of captured samples to streptavidin beads and removal of non-specific material

Bead Wash Buffer, Wash Buffers I, II and III, and Stringent Wash Buffer were diluted to 1× working stocks (SeqCap EZ Hybridization and Wash Kit, Roche NimbleGen). Four hundred microliter of 1× Stringent Wash and 100 μl of 1× Wash Buffer I per capture were aliquoted into tubes and preheated to 47°C and the Capture Beads are allowed to come to room temperature and mixed thoroughly by vortexing, 100 μl of Capture Beads (per capture) were aliquoted into a 1.5 ml centrifuge tube. Beads were placed on a magnetic device and allowed to clear. The Capture Beads were washed twice using 200 μl of Bead Wash Buffer (per capture), vortexed for 10 s, then put on a magnet to allow the solution to clear, and the supernatant discarded. After the second wash, 100 μl of Bead Wash Buffer (per capture) were added to the beads and resuspended. One hundred microliters of resuspended beads were transferred to 0.2 ml PCR tubes and cleared with a magnet. The buffer was discarded and the hybridization samples were transferred to the tubes containing the washed Capture Beads. The hybridization samples with the beads are then incubated for 45 min at 47°C. The samples were vortexed briefly at 15 min intervals to ensure the beads remained in solution. After the 45 min incubation, 100 μl of 47°C 1× Wash Buffer were added to each hybridization sample. Samples were vortexed and the contents transferred to new 1.5 ml centrifuge tubes. The 1.5 ml centrifuge tubes were placed on a magnet and the supernatant was removed. Beads plus bound DNA were washed by adding 200 μl of 1× Stringent Wash Buffer to the beads. Samples were mixed by pipetting up and down then incubated at 47°C for 5 min. Following the 5 min incubation, the sample tubes were placed on a magnet and the supernatant was removed. The 200 μl wash using 1× Stringent Wash Buffer with a 5 min incubation at 47°C was then repeated. After the second wash, the tubes were placed on a magnet and the Stringent Wash Buffer supernatant was removed. Two hundred microliters of 1× Wash Buffer I were added to the beads. Tubes were taken off the magnet and vortexed continuously for 2 min. Tubes were then placed on a magnet and the liquid was removed and discarded. Two hundred microliter of 1× Wash Buffer II were added to the beads. Tubes were taken off the magnet and vortexed continuously for 1 min. Tubes were placed on the magnet and the liquid was removed and discarded. Two hundred microliters of 1× Wash Buffer III were then added to the beads. Tubes were taken off the magnet and vortexed continuously for 30 s. Tubes were placed on the magnet and the liquid was removed and discarded. Finally, the tubes were removed from the magnet and 50 μl of PCR grade water were added to each tube of bead-bound capture sample.

### Post-capture LM-PCR of captured samples

The captured Bisulfite-converted Sample Libraries were amplified using LM-PCR (SeqCap Epi Enrichment Kit, Roche NimbleGen). Twenty microliters of the Captured Library and plus Capture beads were added to two new 0.2 ml PCR tubes containing the Post-Capture LM-PCR Master Mix (25 μl of 2x KAPA HiFi HotStart Ready Mix and 5 μl of 5 μM Post LM-PCR Oligo 1 and 2). Captured samples were amplified in a thermal cycler using the following program: step 1: 98°C for 45 s, step 2: 98°C for 15 s, step 3: 60°C for 30 s, step 4: 72°C for 30 s, step 5: go to step 2, repeat 15 times, step 6: 72°C for 1 min and step 7: 4°C indefinitely. After the completion of the LM-PCR thermal cycler program, the separately amplified, captured bisulfite-converted libraries were pooled by transferring them to a single new 1.5 ml tube containing 500 μl of Qiagen Buffer PBI with the pH indicator added (QIAquick PCR Purification Kit, Qiagen) along with 10 μl of 3M Sodium Acetate. Tubes were vortexed to mix, then centrifuged. The entire volume (∼600 μl) was transferred to a QIAquick Spin Column and centrifuged for 1 min at 13 000 xg. The flow-through was discarded and the column was washed with 750 μl of PE buffer (220 ml of 100% ethanol added to 55 ml of PE). The column was centrifuged for 1 min at ∼13 000 xg. The flow-through was discarded and the column centrifuged a second time to remove residual wash buffer. The column was placed in a new 1.5 ml centrifuge collection tube and 50 μl of pre-warmed PCR grade water were added to the center of the column. The column was centrifuged for 90 s at 13 000 xg. The concentration of the amplified captured bisulfite-converted library was determined using a Nanodrop Spectrophotometer (Thermo Fisher Scientific) and 1 μl of sample was analyzed on a DNA 1000 Chip using a 2100 Bioanalyzer instrument (Agilent Technologies).

### TAB-seq library preparation

The TAB-seq protocol was performed using the commercial kit provided by WiseGene. To monitor the conversion rate, we added spike-in hmC, mC and unmethylated C controls, created by amplifying PCR products with a 5-hmC dNTP mix (Zymo Research), 5-mC dNTP mix (NEB) or dNTP mix (Invitrogen). To generate the 5-hydroxymethylcytosine spike-in control, we followed the protocol described by Yu *et al*. (41). Generation of unmethylated and 5-methylcytosine spike-in controls was performed using Phusion High-Fidelity DNA Polymerases (Thermo) with a total volume of 50 μl (10 μl of 5x Phusion High-Fidelity DNA Polymerases buffer, 1 μl of Phusion High-Fidelity DNA Polymerases, 1 μl of 10 mM 5-methylcytosine or cytosine dNTP Mix, 50 ng of unmethylated lambda DNA (PROMEGA), 2 μl of 10 μM of forward and 2 μl of 10 μM of reverse primers and up to 50 μl of nuclease free PCR grade H_2_0). Thermal cycling conditions were step 1: 95°C for 10 min, step 2: 95°C for 30 s, step 3: 60°C for 30 s, step 4: 72°C for 30 s, step 5: go to step 2, repeat 42 times, step 6: 72°C for 10 min and step 7: 4°C indefinitely. After the PCR amplification, all spike-in controls were subjected to gel extraction to eliminate the template DNA. The primer sequences we used for the spike-in controls are listed in Supplementary Table S3.

### Capture designs

We created five different designs to test the protocol. The capture designs are available as separately downloadable supplementary files.

*Human design 130912_HG19_JG_188_EPI_capture_targets.bed*. Bivalent domains and several contiguous regions.

*Human design 130912_HG19_Methyl_alt_EPI_capture_targets.bed*. This design represents loci interrogated by the Agilent SureSelect MethylSeq platform.

*Human design 130912_HG19_CpGiant_4M_EPI.bed*. The CpGiant catalog design is targeted to compare with the regions represented by the Illumina Infinium HumanMethylation450 microarray.

*Mouse design 131216_MM10_JG_EPI_capture_targets.bed*. We designed custom capture probes to capture 26 392 regions covering ∼24 Mb of mouse genome where DNA modification conserved regions, male and female differentially expressed genes, pluripotent stem cell specific genes and eryhtroid progenitor (EP)-specific genes. While the mouse design was originally based on assembly mm10, we performed our analyses using the UCSC *liftover* function to generate mm9 coordinates, which we provide as the design file in the Supplement to facilitate re-analysis of the data presented here.

*Maize design 130916_Maize_NS_EPI_capture_targets.bed*. A set of regions covering ∼5 Mb of maize genome were selected based on whole genome bisulfite sequencing (WGBS) data of maize inbred lines B73 and Mo17 (42). These regions fall into two major types: regions with or without DNA methylation differences between B73 and Mo17. The regions that do not have DNA methylation differences between B73 and Mo17 can be further divided into three types: all_high, all_low and context-dependent. The all_high regions have a summed DNA methylation level across CG, CHG and CHH contexts for both B73 and Mo17 of at least 4.2 (0–1 scale for each sequence context and each genotype), and the read coverage in both genotypes is at least 85%. The all_low regions are unmethylated regions across all cytosine contexts in both B73 and Mo17, with a read coverage of at least 90%. The context-dependent regions are a list of regions with high DNA methylation levels in particular sequence contexts (CG > 0.95, CHG > 0.2 or CHH > 0.75) in both B73 and Mo17 and relatively lower methylation level in the other sequence contexts (<0.2), with a read coverage of at least 80%. No regions with only CHH DNA methylation were identified but we did select regions that had particularly high CHH levels (>0.75) that also contained DNA methylation in other contexts. The regions that show DNA methylation differences between B73 and Mo17 are divided into three types based on the sequence contexts of the differentially methylated cytosine: CG, CHG or CHH. Some regions may show differential DNA methylation at more than one sequence context.

### Capture probe selection

Probes of variable length, ranging from 50 to 100 nucleotides, were generated at a 5 bp tiling interval across the entire genome, for both top and bottom strands. Probe sequences were *in silico* treated with bisulfite, assuming either all or no CG dinucleotides to be methylated. This created a total of four probe sets, two for each strand. Highly repetitive probes were removed by comparing each probe sequence to a 15-mer frequency table created by *in silico* treatment of the genome, assuming no CGs to be methylated and removing any probe sequence that had an average 15-mer frequency greater than 10 000. Uniqueness of probes in the genome was determined using the whole genome bisulfite sequencing mapping program *bsmap* (43). A slight modification was made to the *bsmap* code to report the number of mapped positions in the genome. Probes that mapped to more than three genomic locations were discarded from further consideration. Probe information, including probe Tm, homopolymer score (based on runs of each base), repeat score and uniqueness were stored in database tables by strand and methylation-state, creating four tables in total. Probes were selected for each region of interest by tiling across each region and selecting all probes within a 15 bp window, at an average spacing of 20 bp between the end of one window and the beginning of the next. On average, 3 probes were evaluated for each 15 bp window, and the best probe was selected based on a rank score calculated based on probe Tm, repetitiveness, uniqueness and homopolymer score. The probe selection process was repeated for each strand and methylation state.

### Massively parallel sequencing

Human and mouse samples were sequenced with the Illumina HiSeq 2500 technology using 100 bp paired end sequencing. Maize samples were sequenced with the Illumina MiSeq technology using either 100 or 150 bp paired end sequencing.

### Sequence analysis

Paired end reads were aligned to the human (hg19) and mouse (mm9) reference genomes using *Bismark* (v 0.10.1; (44)) using *bowtie2* (v 2.1.0) as the underlying alignment software, allowing one mismatch in the 25 bp seed sequence (-N 1 –L 25). Default parameters were used for the remaining settings. After alignment, read duplicates were removed using the *deduplicate_bismark* application included with the *bismark* software distribution. Methylation values were calculated using the *bismark_methylation_extractor* application, ignoring the first two bases on each read (–ignore 2/–ignore_r2 2), and avoiding scoring overlapping methylation calls twice (–no_overlap). For on-target and coverage calculations, the BAM files produced by *Bismark* were first coordinate sorted using *samtools* (v 0.1.19) and overlapping reads were clipped using the *bamUtil* package (https://github.com/statgen/bamUtil). On-target rate was calculated using the *bedtools intersect* command (https://github.com/arq5x/bedtools2) (45), and counting the number of reads which overlap the target regions by at least 1 bp, and dividing by the total number of aligned reads. No padding was added to the target regions for on-target calculations. Mean and median coverage of the target regions were calculated using the *bedtools coverage* command, and summarizing the resulting files using an in-house script. Fold enrichment was determined using Picard's *CalculateHsMetrics* tool (https://github.com/broadinstitute/picard).

For maize, sequencing reads were aligned to the reference genome of maize (version 2) using exactly the same approach as for human and mouse. Reads that mapped to multiple locations were discarded. Uniquely mapped reads were then used to summarize DNA methylation levels at each sequence context (CG, CHG and CHH, where H = A, T or C) for each cytosine again using *bismark_methylation_extractor*. The bisulfite conversion rate of each library was calculated using the cytosine conversion information of the unmethylated chloroplast genome.

### SNP detection

A list of single nucleotide polymorphisms (SNPs) in the maize genome distinguishing the two parent alleles (B73 and Mo17) was compiled from two sources: the maize HapMap2 SNP data (46) and *de novo* called SNPs from the sequencing data of the heterozygous F1 sample. The HapMap2 SNP data were downloaded from Panzea (www.panzea.org/), only retaining the SNPs annotated in both B73 and Mo17 and within the target regions. SNPs were also called from the bisulfite sequencing data of the F1 hybrid using *Bis-SNP* (47) using default parameters. The *de novo* called SNPs that met the following criteria were retained: a quality score of at least 20, and read coverage of ≤120. SNPs within 20 bp of each other were also filtered out. To ensure the quality of SNP data, only the SNPs that were present in the HapMap2 list and the *de novo* called SNP list were used.

### Assignment of reads to parental origin

To distinguish the parental origin of each mapped reads in the heterozygous hybrid (F1), the F1 sequencing reads were first mapped to maize reference genome (version 2) as described above. Uniquely mapped reads were then assigned to one of the two parents (B73 or Mo17) based on the above SNP list using a customized Perl script. Because of the sodium bisulfite treatment, the SNPs that we see in the sequencing of converted DNA do not fully reflect those in the original genomic sequence, if the SNP involves a C on either strand. Such SNPs that became non-informative after bisulfite conversion were discarded. Because of the uneven distribution of SNPs in the genome, some sequencing reads might have more than one SNP while others might have none. For those two situations, we apply the following criteria to distinguish the possible parental origin. For a read lacking SNPs, we checked its mate in the paired end sequencing and assigned it to the same parental allele as the mate if the origin of the mate could be determined. For reads with more than one SNP and any ambiguity regarding parental assignment, we assigned a read to a parental allele if more than 60% of the SNPs present in a read supported that parental origin.

### TAB-seq data analysis

The same alignment approach was used for these data, quality filtering the reads and trimming the first 5 nucleotides of the insert. Reads that are mapped to multiple locations were discarded. Uniquely mapped reads were then used to summarize 5-hydroxymethylation level at CpGs for each cytosine using *methylKit* (48) and *methylation extractor*. Bisulfite conversion efficiencies for each library were calculated using the cytosine conversion rates of unmethylated spike-in controls. We filtered regions where the coverage was <10 and selected cytosines located within the capture target regions (mm9) for analysis extracted by the *intersectBed* command from *bedtools*. We calculated the 5hmC level as unconverted percentage of TAB-seq (41), and the 5mC level by subtracting the unconverted percentage of TAB-seq from the unconverted percentage of BS-seq.

### Testing micro-organismal contamination rates

To measure the proportion of reads from buccal epithelial brushing samples derived from oral micro-organisms, alignment was performed against the NIH Human Microbiome Project (HMP) reference genome database (HMREFG: http://hmpdacc.org/HMREFG/). This reference is described to contain all archaeal, bacterial, lower eukaryote and viral organisms available in GenBank as of November 2009 and reference genomes sequenced as part of the HMP initiative, as well as all other publicly available human associated reference genomes. The database contains 131 archaeal strains over 97 species, 326 lower eukaryotes over 326 species, 3683 viral strains over 1420 species and 1751 bacterial strains over 1253 species. The bacterial component of the database underwent a process of removing highly redundant, non HMP-sequenced reference genomes. The version used in the current project was downloaded on 2 October 2013.

## RESULTS

### Capture performance

We provide a detailed description of the assay, referred to as SeqCap Epi, in the Methods section, with an experimental overview in Supplementary Figure S1. To test capture performance, several different designs were created, targeting human, mouse or maize samples, each capturing different proportions (Supplementary Table S1) and types of sequence features (Supplementary Figure S2) within the genomes tested. Following optimization of conditions for this new assay, we achieved reasonably stable performance characteristics reflected by the results in Supplementary Table S1. This table summarizes the outcomes of multiple different types of experiments performed over time during which the assay was in the final stages of optimization, and includes some relatively less satisfactory outcomes, but we present all of these results in the interests of transparency, to illustrate both the generally reasonable performance of the assay and how it has performed less well on occasion. Including the less successful results, the overall median PCR duplicate rate was <10%, the percentage of on-target reads was ∼53%, the fold enrichment exceeded 85 and conversion efficiency was 99.8%. These values represent capture performance specifications comparable with those that we have published for unmodified DNA in exome-seq assays (49) (Supplementary Table S2), apart from the relatively decreased proportion of on-target reads, which may be due to the relative sequence degeneracy of bisulfite-treated DNA.

We designed an 84 Mb capture system targeting the same human genomic regions as those represented by the SureSelect Methyl-Seq system (Agilent Technologies) and performed triplicate experiments using both systems with DNA from the GM12762 lymphoblastoid cell line (a male subject from one of the CEPH pedigrees). The experimental protocol used for the Agilent SureSelect Methyl-Seq system was that provided by the company with the product. A difference between the systems is that the Agilent system is designed to capture only one DNA strand, whereas SeqCap Epi captures both strands. The on-target rate for the Agilent capture-then-convert strategy was high (88.3–90.3%), but the PCR duplicate rate, based on identity of both start/end positions and DNA methylation patterns within the read, was 25.0–45.7% (Supplementary Table S3).

While this result appears to confirm our concern that this capture-then-convert strategy yields low-complexity libraries, despite starting with the manufacturer's recommended 3.0 μg of DNA, we recognized that the SureSelect Methyl-Seq system only captures one strand of the DNA, and that this increases the tendency to identify reads as PCR duplicates compared with the convert-then-capture approach testing both strands. We therefore took the reads from both the SureSelect Methyl-seq and the convert-then-capture mimic design captures re-analyzed them using *bismark*. We retained only what is called by *bismark* the GA strand for the genome for both data sets. We then intersected each .bam file with the capture targets for the design, and then sub-sampled 7.5 million read pairs for each design. We then used Picard's *MarkDuplicates* to determine the duplicate rate for the sampled reads. The convert-then-capture approach continues to give low PCR duplicate rates ranging from 4.9 to 12.0%, but the SureSelect Methyl-seq data are improved by this analysis focusing on a single strand, now in the range of 16.4–17.5% PCR duplicates (Supplementary Table S4).

### Testing performance with low amounts of input DNA

With the recognition that we were generating high-complexity libraries using the convert-then-capture approach, we wanted to test whether we could limit the amount of starting DNA and retain reasonable library complexity. We used the same NA12762 DNA source for an experiment testing 750 ng, 1.0, 2.0 and 3.0 μg of starting DNA amounts and the *130912_HG19_JG_188_EPI_capture_targets* design, with otherwise identical experimental conditions performed simultaneously. We show in Supplementary Table S5 that the performance for the 750 ng amount of starting material was indistinguishable from the higher amounts of DNA.

We therefore proceeded to try using even more limited amounts of input NA12762 DNA and the same capture design, and present results in Supplementary Table S6, as well as illustrating the PCR duplicate and on-target rate measures of performance from experiments using 1000 down to 10 ng of DNA (Supplementary Figure S3). We find that 500 ng performs as well as 1000 ng in terms of sensitive parameters, but that there is a progressive worsening of performance at and below 100 ng. However, we observe that only when input DNA of 10 ng is used that we fail to generate any data; deeper sequencing of as little as 50 ng of input DNA can be used to generate enough coverage for quantitative measurement of DNA methylation in the regions captured.

### Capture reproducibility

**Figure 1.**
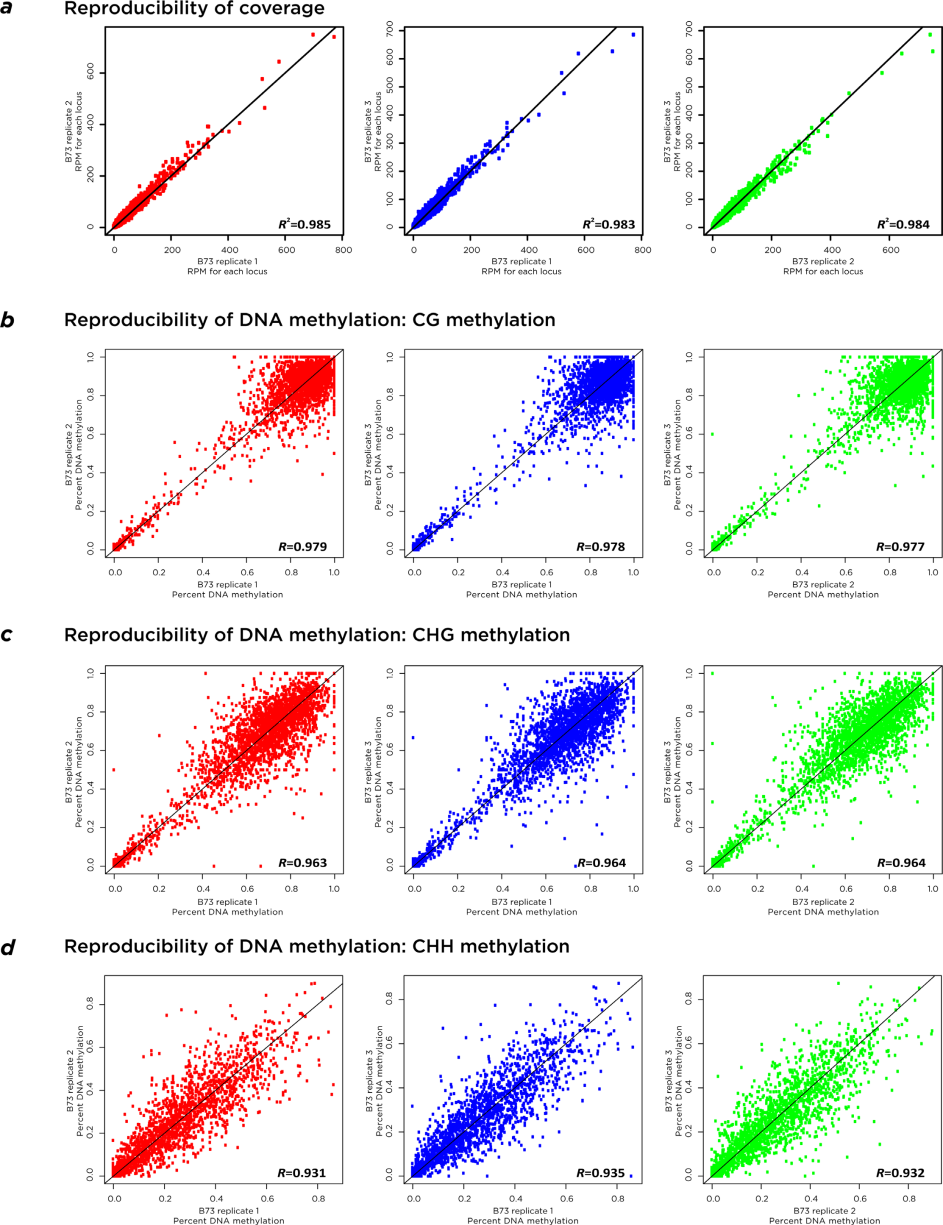
Assay reproducibility. We show three replicates of the capture assay performed on the same sample of maize genomic DNA. In panel (**a**), the reproducibility of coverage is shown to be very consistent. Panels (**b–d**) show reproducibility of DNA methylation in CG, CHG and CHH contexts, all showing comparable and high degrees of concordance of values. The values shown are for over 4000 genomic loci each of sizes 300–1000 bp.

We illustrate the results of capture reproducibility from experiments using our maize samples. In Figure 1a we show read coverage reproducibility among three technical replicates of the B73 inbred maize line, reproducing the experiments from the same DNA sample and plotting the read number as reads per million sequences. Red dots are between replicate 1 and replicate 2; blue for replicates 1 and 3; green for replicates 2 and 3. The plot illustrates the highly concordant coverage between replicates.

In Figure 1b–d we show the reproducibility of DNA methylation from the same samples. Because we used maize for these comparisons, we were able to test not only the reproducibility of CG but also of CHG and CHH methylation in the same B73 samples. The reproducibility of DNA methylation is high in all cases. We observe bimodality to CG and CHG DNA methylation, with loci that are both unmethylated and extensively methylated, and a tendency of CHH cytosines to be less methylated.

In Supplementary Figures S4 and S5, we show the same plots but color coded for different genomic and base composition contexts, demonstrating that there are no obvious problems with reproducibility in any of these subsets of loci.

### Testing for bias in capture of DNA methylation states

To test for bias in capture of methylated or unmethylated DNA, we performed two analyses. In Supplementary Figure S6 we show the result of testing coverage of CG dinucleotides in each decile of DNA methylation. Any tendency to capture methylated or unmethylated DNA preferentially should be reflected by a trend in these distributions, but no such trend is observed.

As a more rigorous experimental test, we used DNA from the grossly hypomethylated HCT116 DKO cell line as the substrate for M.SssI methylase for a treatment lasting 60 min. The untreated and 60 min treatment samples were captured separately and in a 50:50 mixture using the *130912_HG19_JG_188_EPI_capture_targets* design. We generated histograms (Supplementary Figure S7) showing the distributions of 5mC in each sample, confirming the low DNA methylation in the untreated HCT116 DKO sample and its conversion to highly methylated DNA following M.SssI methylase treatment. We then performed a simulation experiment in which we sampled data from the untreated and 60 min treated samples in equal proportions, showing a histogram representation of the distributions of DNA methylation expected by the simulation results and observed using the capture results. Any systematic bias in favor of capturing methylated or unmethylated DNA should result in a deviation of the observed from the expected distribution, but we instead find the distributions to be highly comparable, allowing us to conclude that the system does not favor the capture of one methylation state over the other.

### Comparison with WGBS data

**Figure 2.**
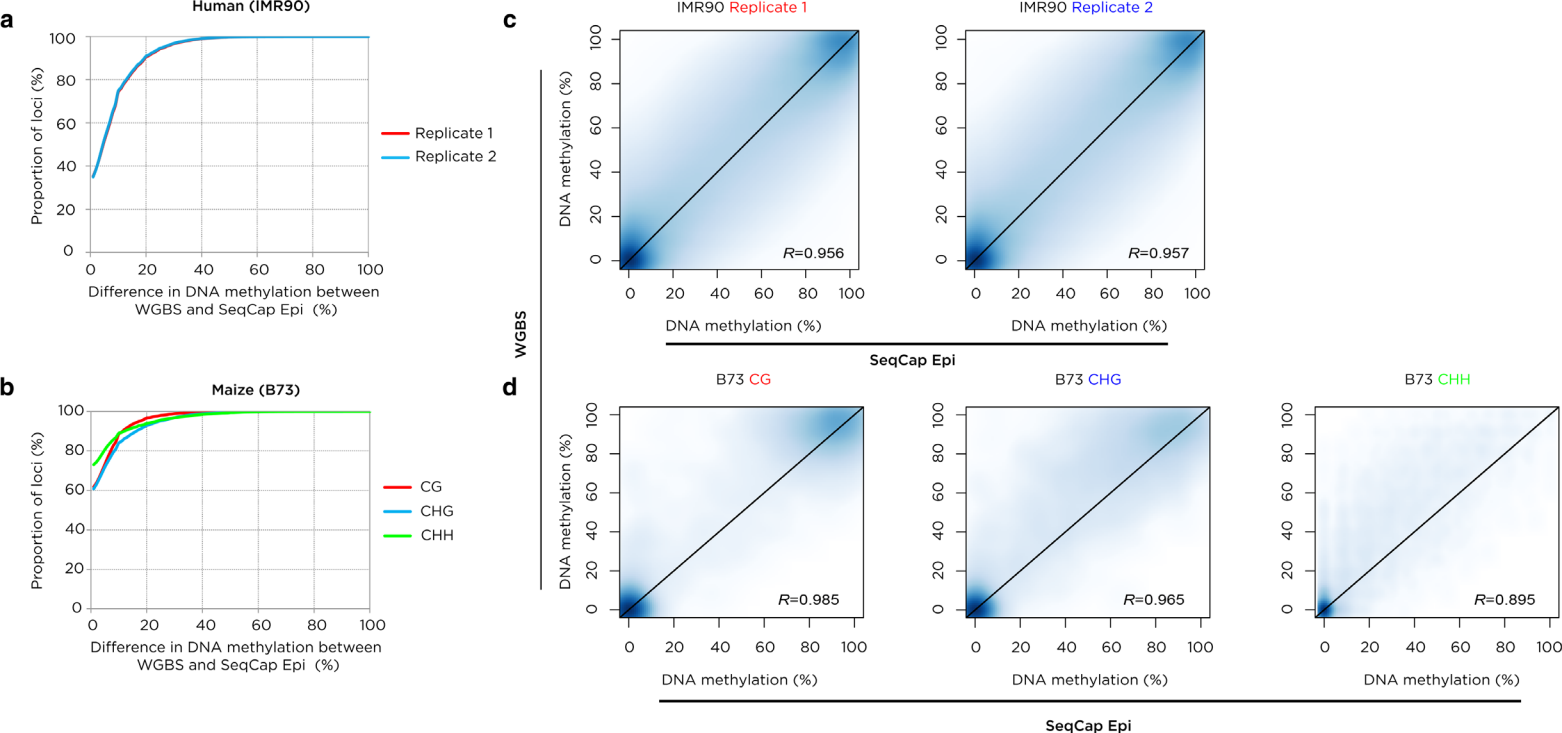
Comparison of capture results with WGBS. In panels (**a**) and (**b**), we show the proportion of loci with different degrees of concordance of DNA methylation values for human (IMR90 cells) (a) and maize (B73) (b) samples. The associated scatter plots showing the correlation of per cytosine methylation values for WGBS and SeqCap Epi are shown in panels (**c**) and (**d**).

We used the IMR90 human fibroblast line in our studies because of the availability of publicly available WGBS data (15) for comparison. The sequencing data being compared are not identical, as the published WGBS used 36 bp single end Illumina GAII sequencing whereas we performed 100 bp paired end Illumina HiSeq 2500 sequencing. However, we see a strong concordance between values genome-wide for two replicates of the capture-based approach, and even greater concordance for the maize genome for which we generated parallel, technically comparable WGBS data (Figure 2).

### Allelic DNA methylation identification using SNP information

**Figure 3.**
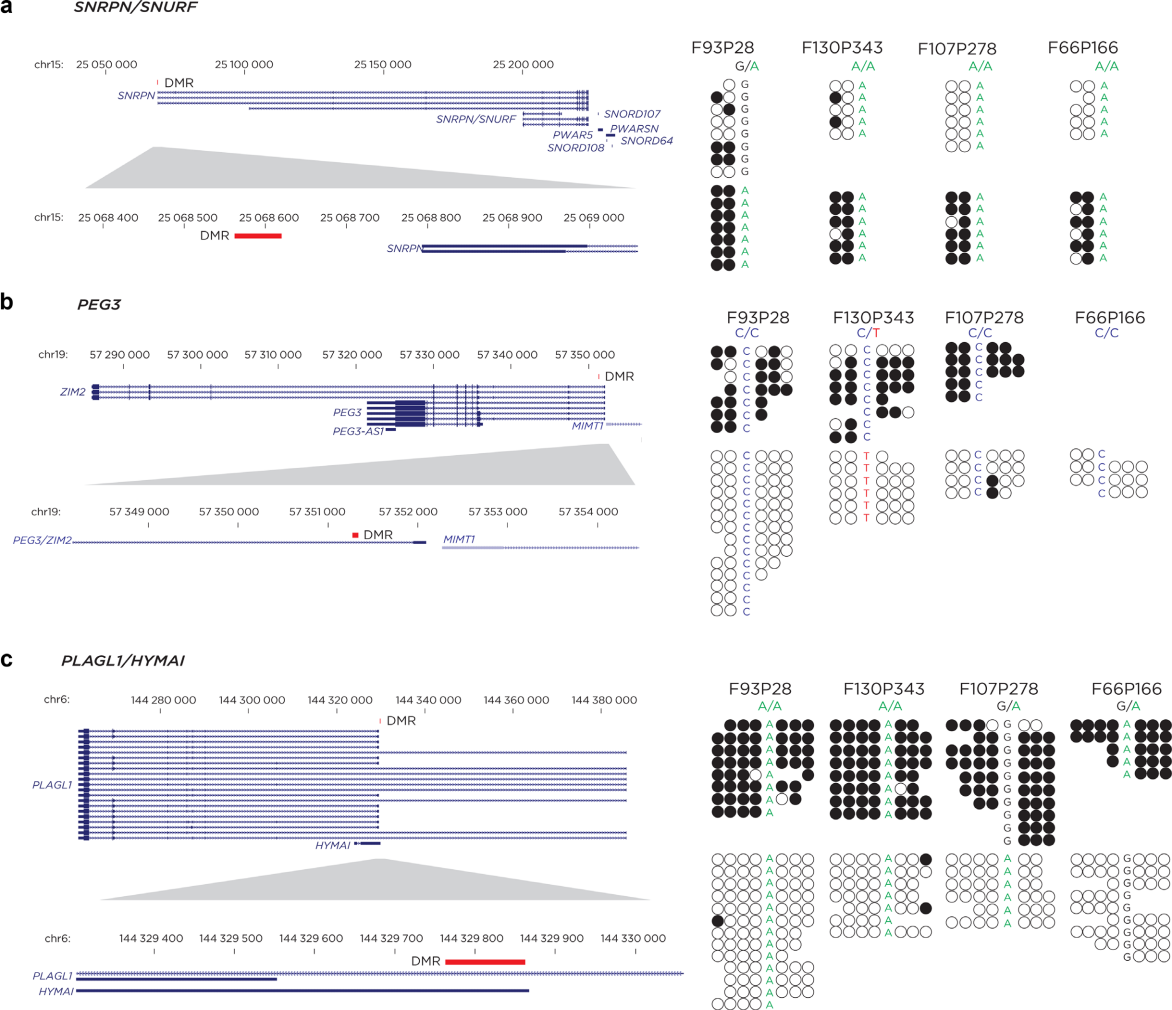
Detection of allelic DNA methylation at imprinted DMRs. Buccal epithelial samples were used in this analysis. We show reads from only one strand for clarity. The reads were distinguished at these loci by intra-read concordance of DNA methylation states, separating into groups of reads that are either very methylated or very unmethylated, sometimes distinguishable by the presence of a heterozygous SNP within the reads that revealed their origins to be from the different parental alleles. This is the pattern expected for imprinted DMRs, at which paternally and maternally derived alleles have distinctive DNA methylation.

We used DNA from buccal epithelial brushing samples that have been previously described (39). Because of the microbial DNA present in buccal brushings, sequencing-based assays are generally not practical, but the capture of human DNA using the SeqCap Epi system was associated with the recovery of 84–95% of sequences that mapped to the human genome when combined with mapping to the human microbiome reference sequence (http://hmpdacc.org/HMREFG/). We used *Bis-SNP* (50) to identify SNPs in the bisulfite sequence data. Several imprinted differentially methylated regions (DMRs) included in the capture design showed the expected patterns of allelic DNA methylation, in some individuals including an informative heterozygous SNP distinguishing the parental alleles (Figure 3). The SeqCap Epi assay is therefore capable of the detection of SNPs and allelic DNA methylation at captured regions, and can be used with DNA samples contaminated by microbial or other non-target DNA.

The capture-then-convert (Agilent SureSelect Methyl-Seq) system only captures one strand, which leads to an insensitivity of detection of certain sequence variants, as we show in Supplementary Figure S8. The capture of both strands allows such loci to remain informative.

### Detection of DMRs

**Figure 4.**
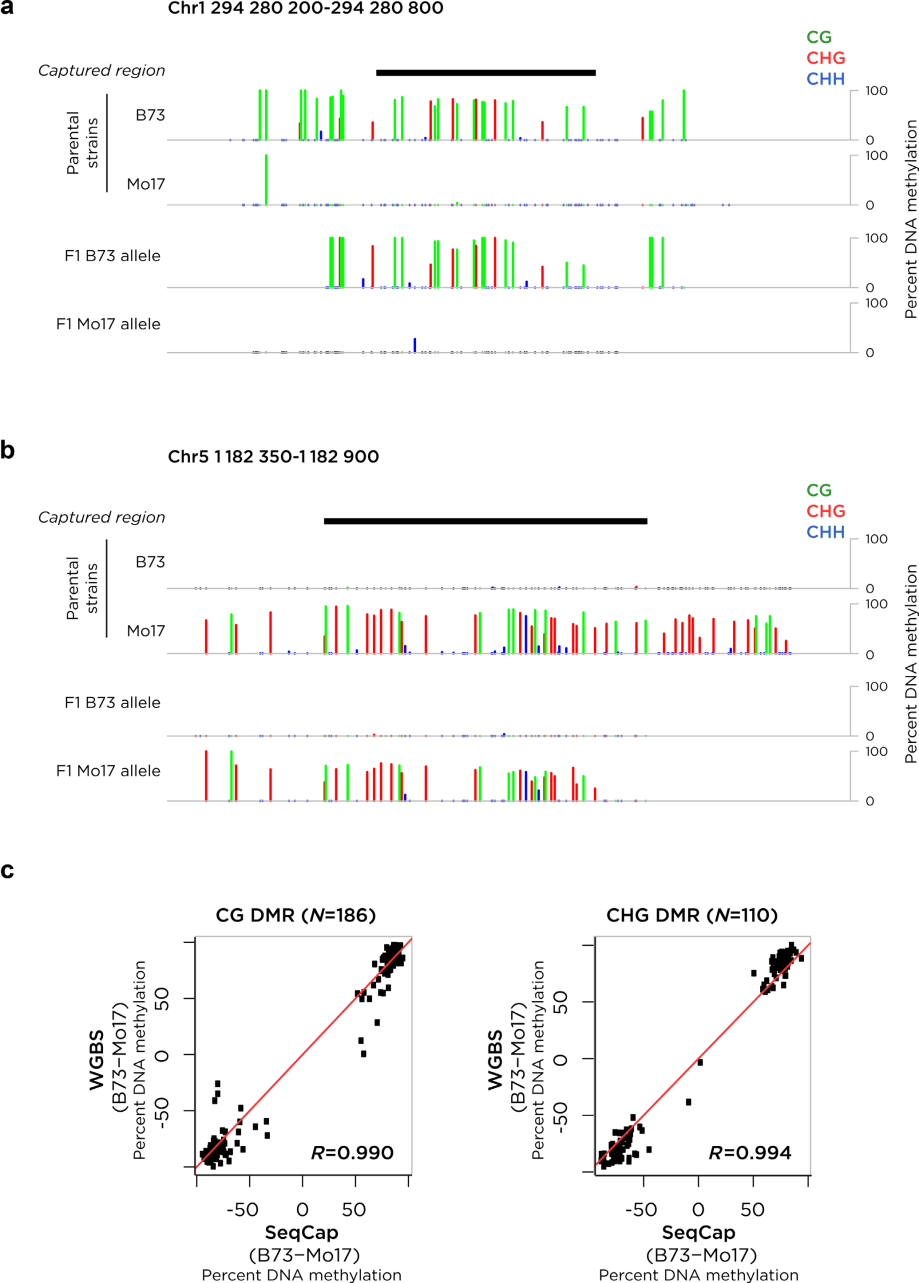
DMRs identified by WGBS are also detected by the capture assay. Two maize strains (B73 and Mo17) were studied. A number of loci with differential DNA methylation were identified by WGBS between the two strains, with two represented in (**a**) and (**b**). In the B73 x Mo17 F1 cross, these DMRs persisted as allelic differences in DNA methylation, distinguished by SNPs (not shown) and including DNA methylation in CG, CHG and CHH contexts, as color-coded. In panel (**c**) we show that the degree of difference of DNA methylation at these DMRs is comparable between the WGBS and capture-based approaches.

The maize data included both WGBS and capture-based experiments on two parental maize strains B73 and Mo17 and on their F1 hybrid. Loci identified as DMRs between the parent lines were included on the capture design, allowing us to assess allelic DNA methylation patterns in the heterozygous F1 plants. We show in Figure 4 examples of two loci where SNPs present in the bisulfite reads allowed us to distinguish the alleles in the F1 sample, revealing allelic differences in DNA methylation in CG, CHG and CHH contexts. We extend this analysis to show how all of the DMRs identified using WGBS compare in terms of the difference in DNA methylation observed using the capture-based approach, showing concordance for the 186 loci with differential CG and the 110 loci with differential CHG methylation (c).

### Detection of 5-hydroxymethylcytosine using TAB-seq and SeqCap Epi

The output of bisulfite sequencing is not merely 5-methylcytosine (5mC) but also includes any 5-hydroxymethylcytosine (5hmC) present, as both modified nucleotides resist bisulfite mutagenesis (51). While we have referred to the output of the bisulfite sequencing up to this point as ‘DNA methylation’, more correctly it should be defined as the sum of 5mC and the smaller proportion of 5hmC at a locus, or 5(h)mC. We tested whether we could discriminate the 5hmC subset of alleles from the 5(h)mC total using the Tet-assisted bisulfite sequencing (TAB-seq) approach (24). TAB-seq involves conjugating a glucose to 5hmC using ß-glucosyltransferase (ßGT). The resulting ß-glucosyl-5-hydroxymethylcytosine (5gmC) is protected from oxidation by recombinant Tet1, whereas 5mC undergoes oxidation to 5-carboxylcytosine, which is converted to carboxyluracil with bisulfite mutagenesis, which also converts unmodified cytosine to uracil. Subsequent sequencing reads both carboxyluracil and uracil as thymine, allowing the remaining cytosines in the sequenced DNA to be detected as having originally been 5hmC.

**Figure 5.**
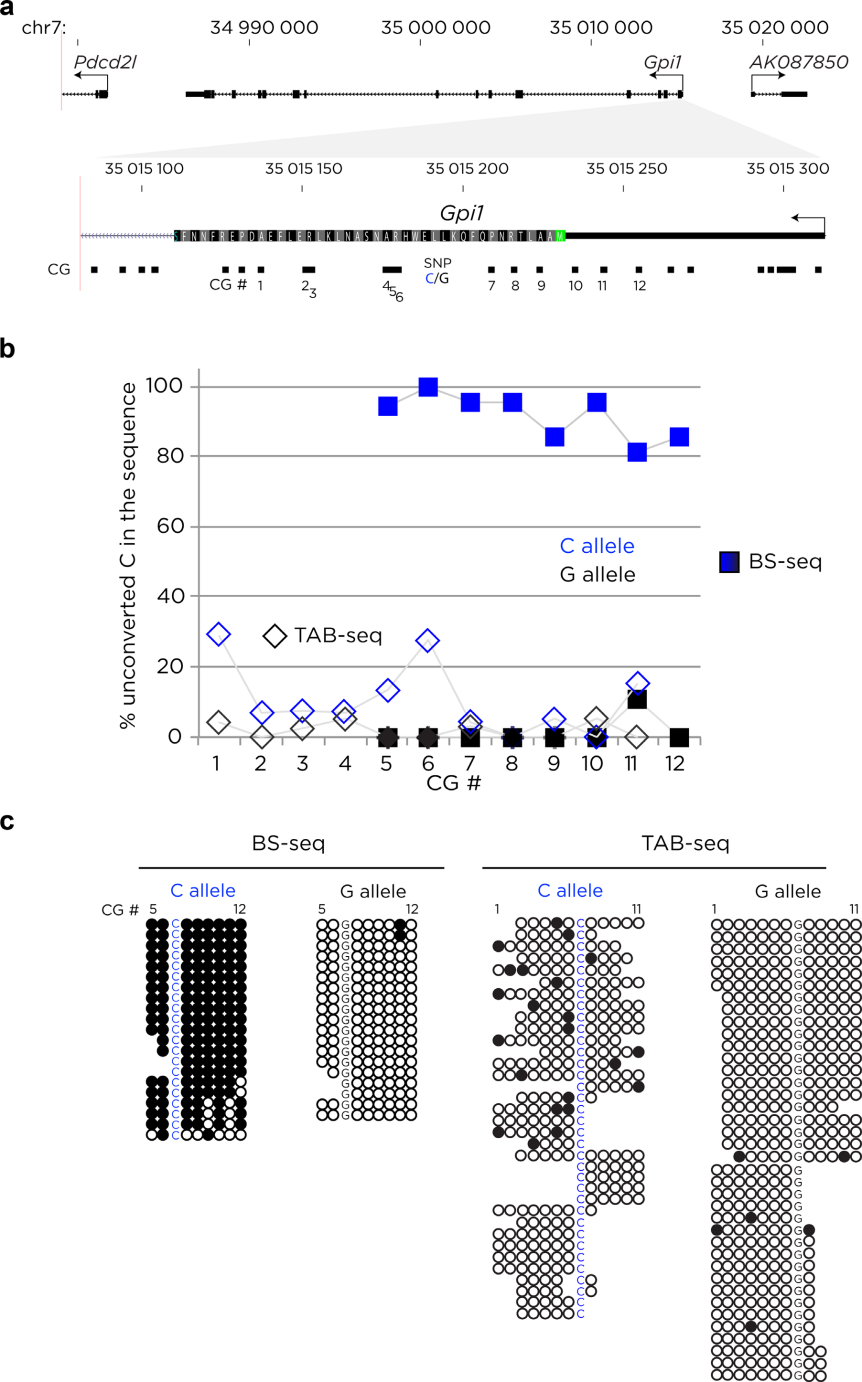
Targeted 5-hydroxymethylation detection and quantification. DNA from mouse ES cells was treated with a standard bisulfite approach and with the TAB-seq protocol to reveal the subset of loci with 5-hydroxymethylation. We show representative results from the Gpi1 locus (**a**), where we found strongly allelic patterns of 5(h)mC from the bisulfite sequencing (BS-seq, squares in (**b**), individual reads shown on left of (**c**)). The results of TAB-seq to detect and quantify 5hmC show that the C allele is more hydroxymethylated than the G allele, demonstrating that the capture approach used downstream of TAB-seq can discriminate allelic hydroxymethylation events.

We performed TAB-seq to detect 5hmC on mouse ES cell DNA from the E14.Tg2a line (52). The spike-in control sequences used are described in Supplementary Table S7. We show in Figure 5 the results of sequencing at one of the captured loci in the mouse genome. Regular bisulfite sequencing shows an SNP distinguishing the differentially methylated alleles at the *Gpi1* gene, which has not been described to undergo genomic imprinting. However, as bisulfite sequencing does not discriminate between 5mC and 5hmC but represents the sum of both sets of modifications (5(h)mC), the TAB-seq data are necessary to allow the subset of 5hmC-modified alleles to be discriminated. We show that the G allele at this locus is not only enriched for 5(h)mC, it is also associated with increased 5hmC levels, which contribute a small proportion of the total 5(h)mC content.

## DISCUSSION

Our results show that a convert-then-capture approach for targeted bisulfite sequencing works robustly in multiple different situations. We tested the capture of different proportions of the genome in different organisms, in which the types of DNA methylation differ, with the mammalian genomes predominantly CG methylated but the maize genome including a greater proportion of CHG and CHH methylation. Compared with the current gold standard for DNA methylation, WGBS, this new assay (commercialized as SeqCap Epi (Roche-NimbleGen)) works robustly, while comparison with the capture-then-convert approach showed that strategy to work better in terms of on-target reads but to have a very high proportion of PCR duplicates in the resulting sequence data, indicating a problem with low library complexity.

For human disease studies testing 5mC variability, practical problems can include limitations in cell numbers and the presence of contaminating sources of DNA from epithelial samples interfacing with the colonizing microbiome. Advances in the optimization of the RRBS assay have allowed the input DNA amount to be reduced to 100 ng (53), facilitating the application of that assay to clinical samples. The performance of SeqCap Epi appeared to be unaltered when starting DNA amounts were reduced to 500 ng and retained the ability to generate on-target reads with as little as 50 ng of input DNA. The targeted capture component of SeqCap Epi allowed us to use buccal brushing samples in a sequencing-based assay, which is normally not possible for survey assays like RRBS or HELP-tagging, as a substantial proportion of reads is derived from contaminating micro-organismal DNA.

We also show the value of SeqCap Epi for studies of 5hmC. It should be noted that the alternative capture-then-convert approach followed by TAB-seq is unlikely to work, as TAB-seq involves causing pre-methylated adapters to be oxidized and mutagenized. The proportion of 5hmC to 5mC in the genome is low, so that the overall allelic contribution of 5hmC modifications will be likewise limited, requiring extremely deep sequencing if we are to measure this DNA modification accurately. Recognizing this, a reduced representation approach has previously been employed as a survey approach allowing deeper sequencing for 5hmC at a subset of genomic loci (25,54). The SeqCap Epi approach allows targeting of loci outside the short MspI fragments used for RRBS with similar quantitative, nucleotide-resolution results. As assays are developed for sequencing of other, even less abundant cytosine modifications (51), the need to sequence to even greater depth using a survey approach will be of even more pronounced value.

The capacity of WGBS and targeted approaches to identify SNPs within the bisulfite-converted reads is of value in identifying allelic DNA methylation, at imprinted loci and at loci subject to the influence of mQTLs. As it is now increasingly apparent that mQTLs exert a very substantial influence upon DNA methylation (55–57), the identification of SNPs encoding potential mQTLs is now an increasingly important part of sequencing-based DNA methylation studies. SNP detection also allows detection of polymorphism in the cytosine being tested. As 5mC is unusually prone to mutagenesis through spontaneous deamination to thymine (58), the sites being tested for DNA methylation carry an attendant risk of being polymorphic at the sequence level. A cytosine transition to thymine at a CG dinucleotide results in a TG dinucleotide, which in bisulfite-converted DNA could be interpreted as an unmethylated cytosine. To resolve this, having sequence information from the other strand will reveal the complementary CA dinucleotide in the situation of a C→T transition on the tested strand. A targeted approach that interrogates both strands has therefore some advantages over an assay testing only one strand.

The performance characteristics of the SeqCap Epi assay have allowed us to gain insights into the amount of sequencing required, and thus a sense of the costs involved. Our experience with a design testing ∼80 Mb of the human genome, performing 100 bp paired end sequencing with the Illumina HiSeq 2500 platform, is that we can exceed mean 30× coverage routinely with three separately indexed samples combined in each lane, with the performance characteristics described in Supplementary Table S1. These sequencing requirements are reasonably comparable with those for the more commonly used exome-seq assay, so the sequencing costs should also be in the same range. In situations when the desired mean coverage or the amount of genome targeted differs, the amount of multiplexing of samples will vary and will influence the cost estimate accordingly.

## ACCESSION NUMBERS

The human data are archived under accession number SRP049215, the mouse data under accession number SRP049154, and the maize data under the following accession numbers: B73 (SRX729949, SRX731435, SRX731436), Mo17 (SRX731440, SRX731441), B73XMo17 (SRX731662, SRX731663, SRX731664).

## SUPPLEMENTARY DATA

Supplementary Data are available at NAR Online.

SUPPLEMENTARY DATA
