# Supplementary material for: Post-conversion targeted capture of modified cytosines in mammalian and plant genomes
Source: Nucleic Acids Res. 2015 Mar 26;43(12):e81. doi: 10.1093/nar/gkv244 (PMC4499119; doi:10.1093/nar/gkv244)
Supplement: SUPPLEMENTARY DATA [file supp_43_12_e81__index.html]

Post-conversion targeted capture of modified cytosines in mammalian and plant genomes — Post-conversion targeted capture of modified cytosines in mammalian and plant genomes — SUPPLEMENTARY DATA 

# Post-conversion targeted capture of modified cytosines in mammalian and plant genomes

## SUPPLEMENTARY DATA

**Files in this Data Supplement:**

- SUPPLEMENTARY DATA
- SUPPLEMENTARY DATA
- SUPPLEMENTARY DATA
- SUPPLEMENTARY DATA
