# Supplementary material for: Post-conversion targeted capture of modified cytosines in mammalian and plant genomes
Source: Nucleic Acids Res. 2015 Mar 26;43(12):e81. doi: 10.1093/nar/gkv244 (PMC4499119; doi:10.1093/nar/gkv244)
Supplement: SUPPLEMENTARY DATA [file supp_gkv244_nar-03338-met-k-2014-File010.pdf]

Post-conversion targeted capture of modified cytosines in mammalian and plant genomes.

Qing Li,[1,*] Masako Suzuki, [2,*] Jennifer Wendt,[3] Nicole Patterson,[2] Steven R. Eichten,[1] Peter J. Hermanson,[1] Dawn Green,[3] Jeffrey Jeddeloh,[3] Todd Richmond,[3] Heidi Rosenbaum,[3] Daniel Burgess,[3,†] Nathan M. Springer,[1,†] John M. Greally[2,†]

[1]Department of Plant Biology, University of Minnesota, 1445 Gortner Ave, Saint Paul, MN 55108

[2]Center for Epigenomics and Division of Computational Genetics, Department of Genetics, Albert Einstein College of Medicine, 1301 Morris Park Avenue, Bronx, NY 10461

[3]Roche-NimbleGen, 500 South Rosa Road, Madison, WI 53711

*co-first authors

†co-corresponding authors

Correspondence and requests for materials should be addressed to daniel.burgess@roche.com, springer@umn.edu or john.greally@einstein.yu.edu

SUPPLEMENTARY MATERIAL

Table of contents:

Supplementary Figures

PAGE

**2**	**Supplementary Figure S1**	Workflow overview.
**3**	**Supplementary Figure S2**	Genomic contexts represented by human and mouse capture designs.
**4**	**Supplementary Figure S3**	Low input DNA performance metrics.
**5**	**Supplementary Figure S4**	Reproducibility of maize DNA methylation in different genomic contexts.
**6**	**Supplementary Figure S5**	Reproducibility of maize DNA methylation in different base compositional contexts.
**7**	**Supplementary Figure S6**	Coverage by deciles of DNA methylation values.
**8**	**Supplementary Figure S7**	Testing HCT116 DNA for evidence of preferential capture of DNA methylation states.
**9**	**Supplementary Figure S8**	An example of the insensitivity of capturing one strand for DNA sequence variant detection.

SUPPLEMENTARY FIGURE S1

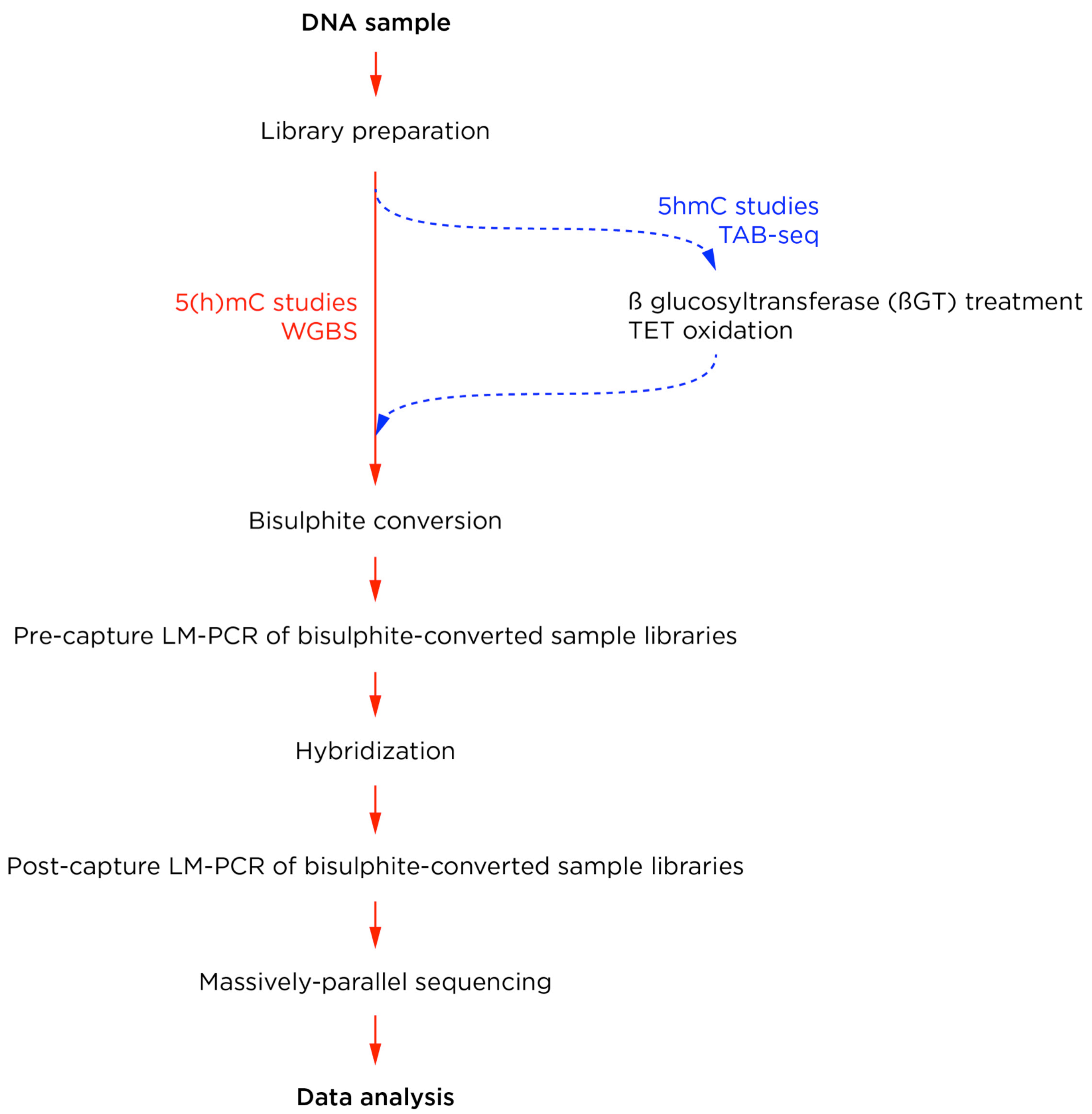

SUPPLEMENTARY FIGURE S1: An overview of the workflow for the capture-then-convert (SeqCap Epi) protocol, with the TAB-seq variation for 5hmC measurement also shown.

SUPPLEMENTARY FIGURE S2

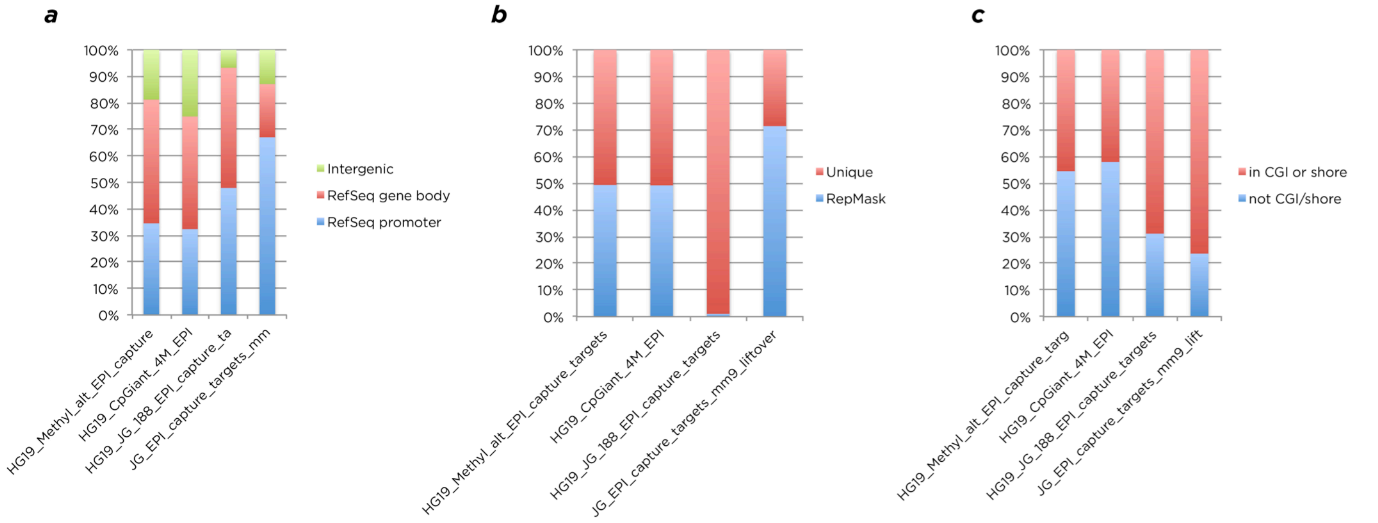

SUPPLEMENTARY FIGURE S2: The capture designs used for human and mouse differed in terms of representations of (a) sequences at RefSeq promoters (transcription start site ± 2 kb) and the remaining RefSeq gene bodies or intergenic regions, (b) unique sequence or those overlapping Repeatmasker annotations, and (c) CpG island or the flanking ±2 kb (shores) or more distant loci. A large diversity of loci and capture designs was thus tested.

SUPPLEMENTARY FIGURE S3

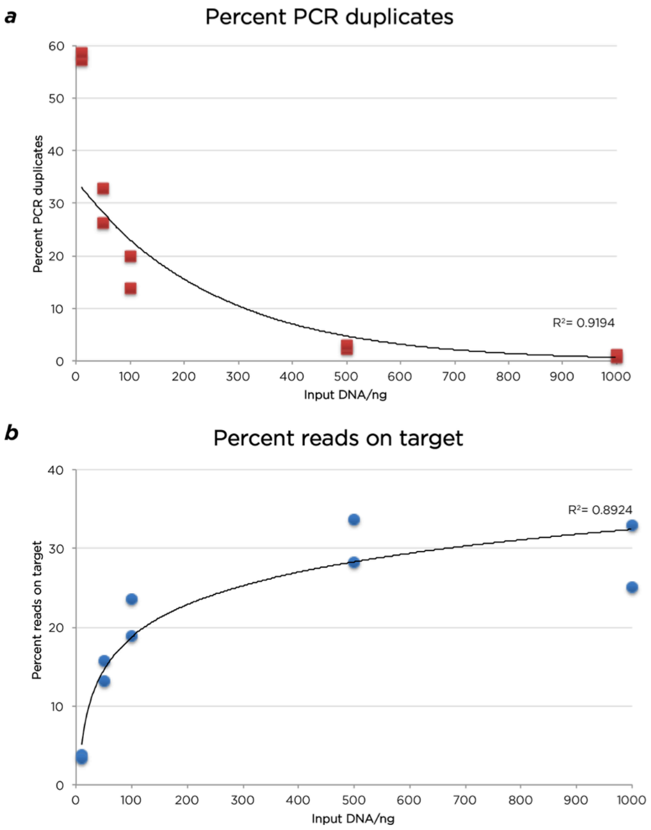

SUPPLEMENTARY FIGURE S3: Low input amounts were tested using the NA12762 sample. We found that performance at 500 ng was indistinguishable from 1,000 ng in terms of PCR duplicates and proportional on target reads, with reduced quality for both of these parameters as input was progressively decreased to 100, 50 and 10 ng, as shown. The data fit exponential (top) and logarithmic (bottom) curves reasonably well (R^2 values shown). While deeper sequencing would be required to get the same number of non-duplicated, on-target reads as for larger input amounts, in situations of scarce material it appears that as little as 50 ng input should allow DNA methylation profiles to be generated.

SUPPLEMENTARY FIGURE S4

a **Reproducibility of DNA methylation in different genomic contexts: CG methylation**

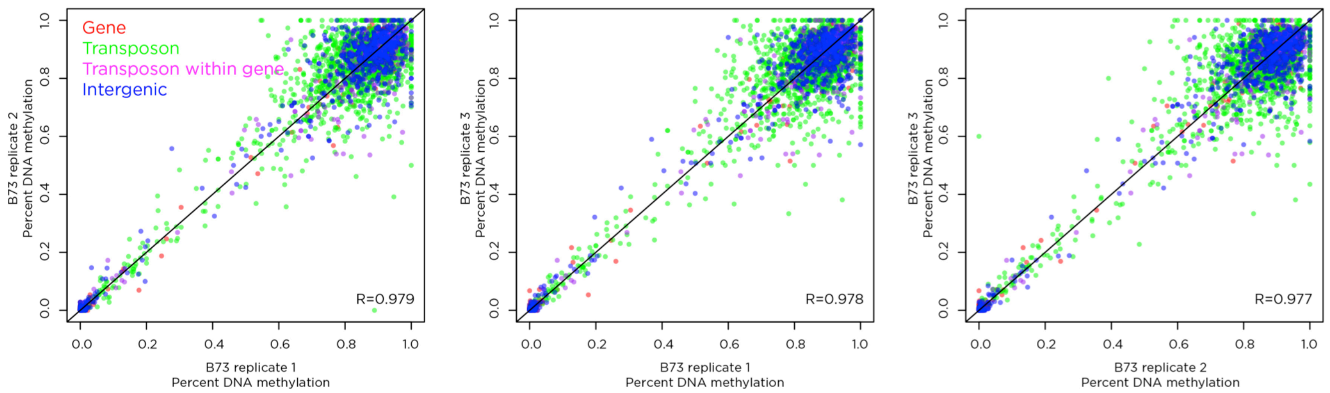

b **Reproducibility of DNA methylation in different genomic contexts: CHG methylation**

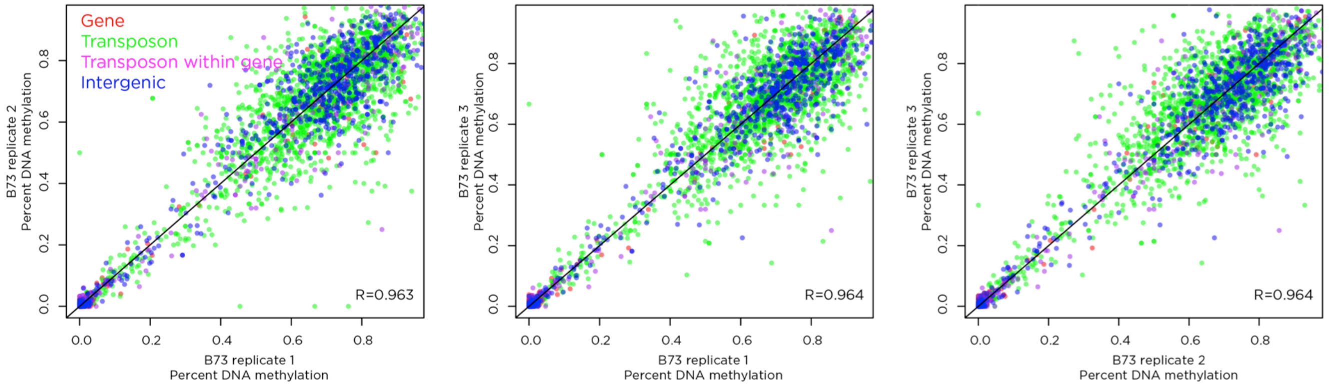

c **Reproducibility of DNA methylation in different genomic contexts: CHH methylation**

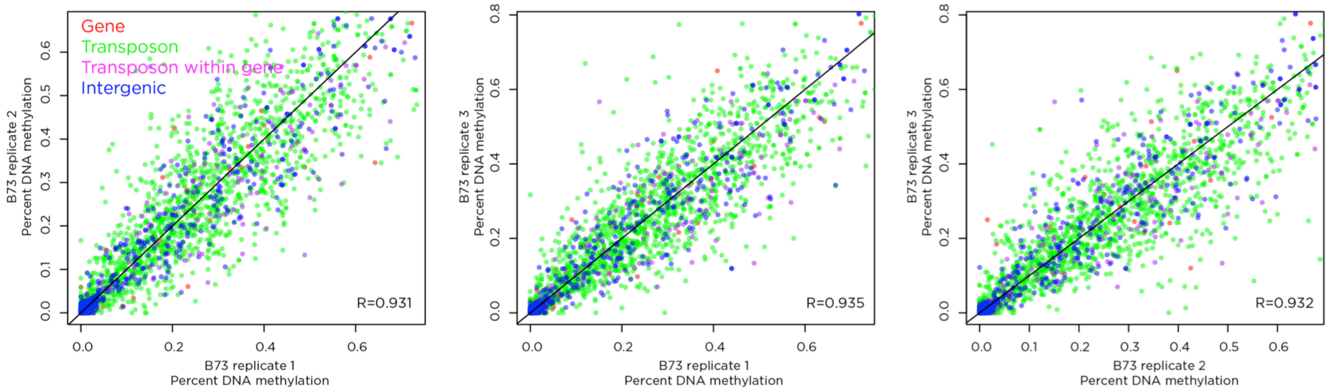

SUPPLEMENTARY FIGURE S4: The maize data were used to allow testing of reproducibility of (a) CG, (b) CHG and (c) CHH DNA methylation within different genomic contexts: genes (red), transposons (green), transposons within genes (pink) and intergenic sequences (blue). The overall R scores are shown, while the color coding allows it to be shown that there are no obvious problems of reproducibility of DNA methylation for any specific genomic context.

SUPPLEMENTARY FIGURE S5

a **Reproducibility of DNA methylation in different base composition contexts: CG methylation**

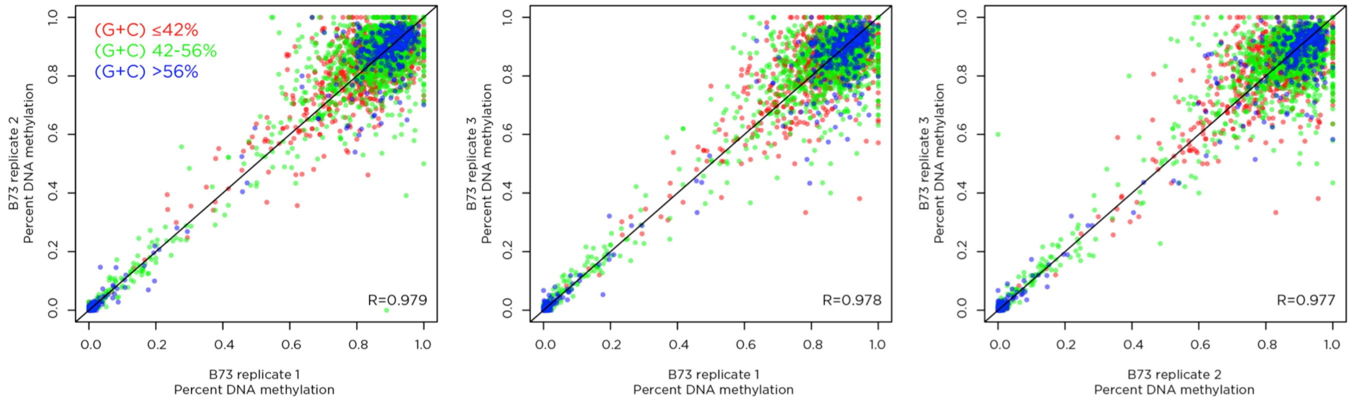

b **Reproducibility of DNA methylation in different base composition contexts: CHG methylation**

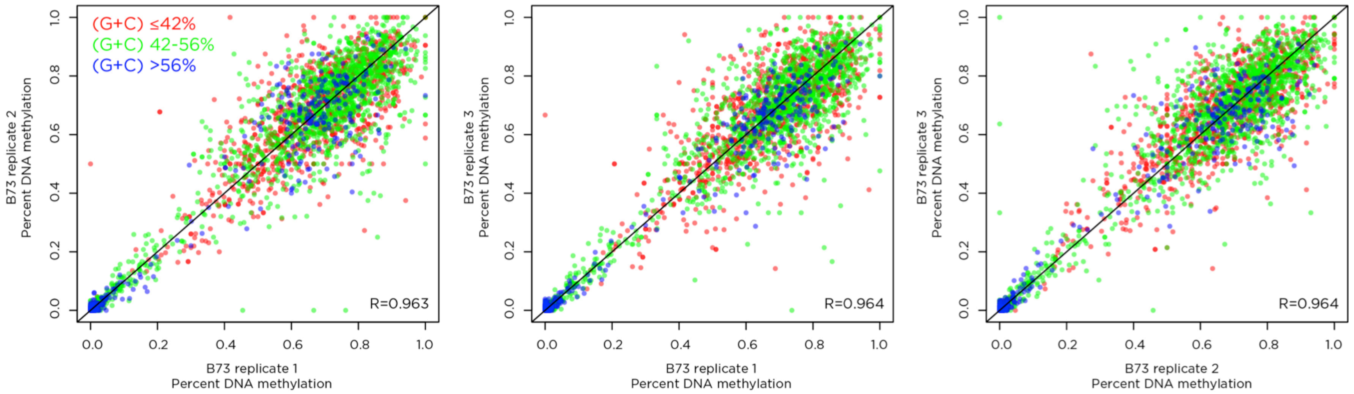

c **Reproducibility of DNA methylation in different base composition contexts: CHH methylation**

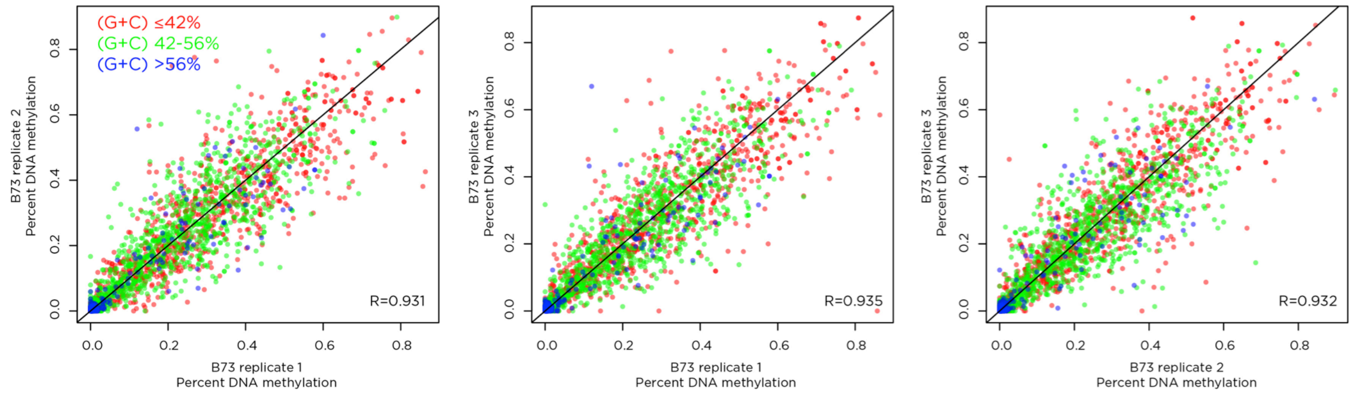

SUPPLEMENTARY FIGURE S5: As for the previous figure, but with different (G+C) mononucleotide contents color coded. Again, no obvious problems of reproducibility of DNA methylation are apparent for any base compositional context.

SUPPLEMENTARY FIGURE S6

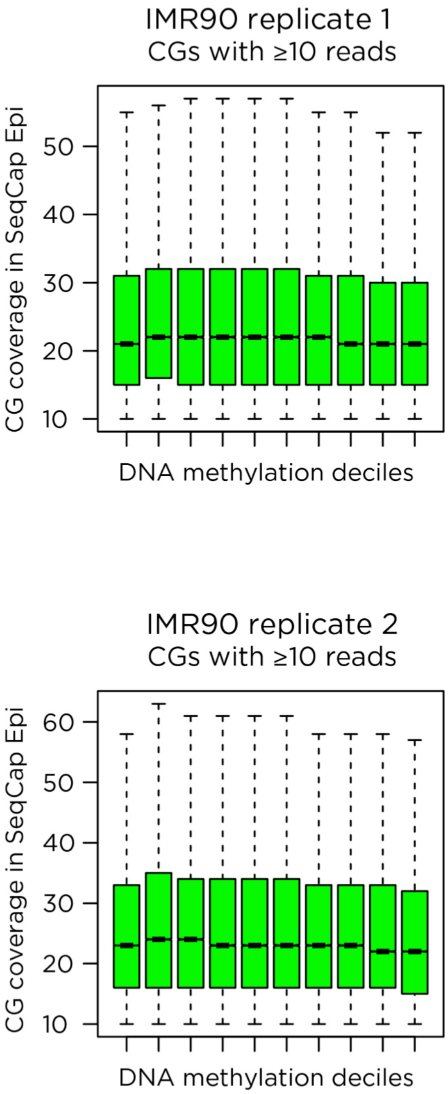

SUPPLEMENTARY FIGURE S6: We show the coverage distributions for deciles of DNA methylation values, at CG dinucleotides with at least 10X coverage. Any bias towards the capture of methylated compared with unmethylated DNA should be reflected by a trend in these distributions, but they appear to be equivalent in each decile for each replicate of the IMR90 capture.

SUPPLEMENTARY FIGURE S7

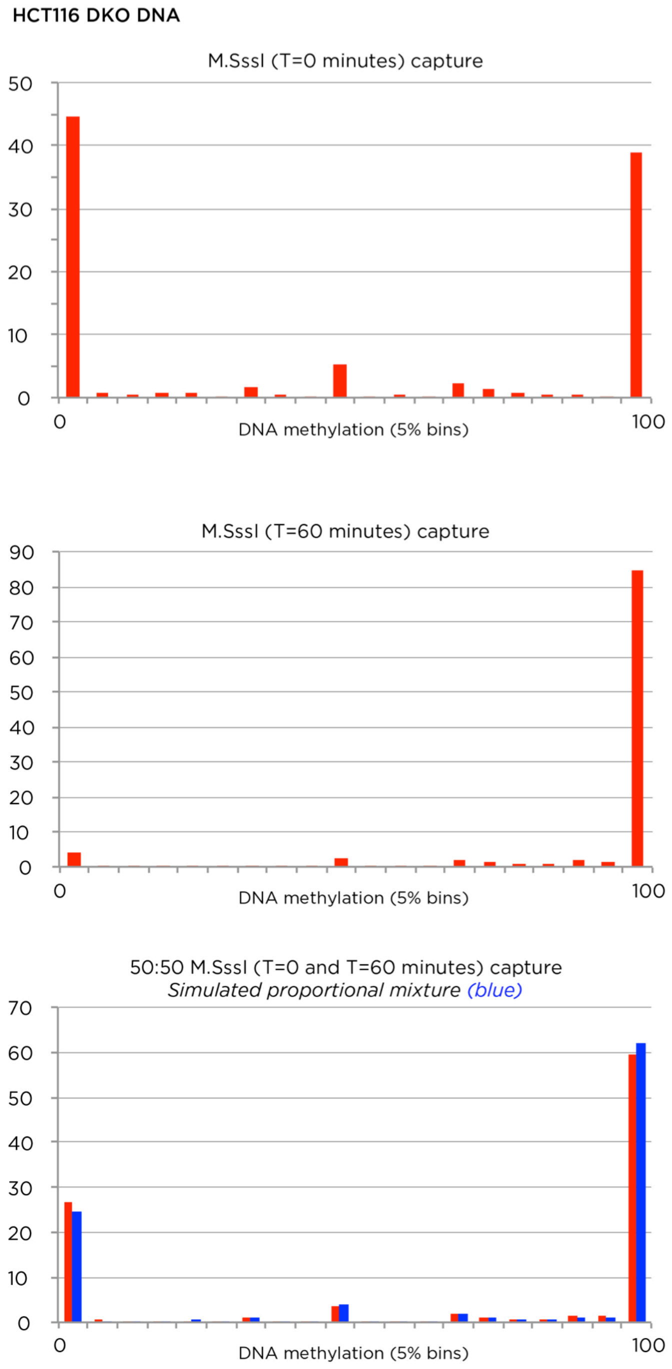

SUPPLEMENTARY FIGURE S7: DNA from the 5mC-depleted HCT116 DKO cell line (Zymo Research) was left untreated (top) or was treated with M.SssI methylase for 60 minutes (middle), or mixed in equal amounts prior to capture with the human 2.8 Mb *130912_HG19_JG_188_EPI_capture_targets* design. The DNA methylation values of cytosines are shown as 5% bins in the histograms, revealing the expected large proportion of unmethylated loci in the untreated sample (top), and near-complete DNA methylation in the sample treated for 60 minutes (middle). In the bottom panel, we show in red the distribution of DNA methylation values resulting from capture of the mixed untreated and treated samples, and compare this with an equal sampling of data from the separate captures of these samples. Any systematic bias in favor of capturing methylated or unmethylated DNA should be reflected by skewing of the red distribution compared with that shown in blue in the bottom panel. As can be seen, the observed results closely match the predicted distributions, showing no evidence for systematic capture bias.

SUPPLEMENTARY FIGURE S8

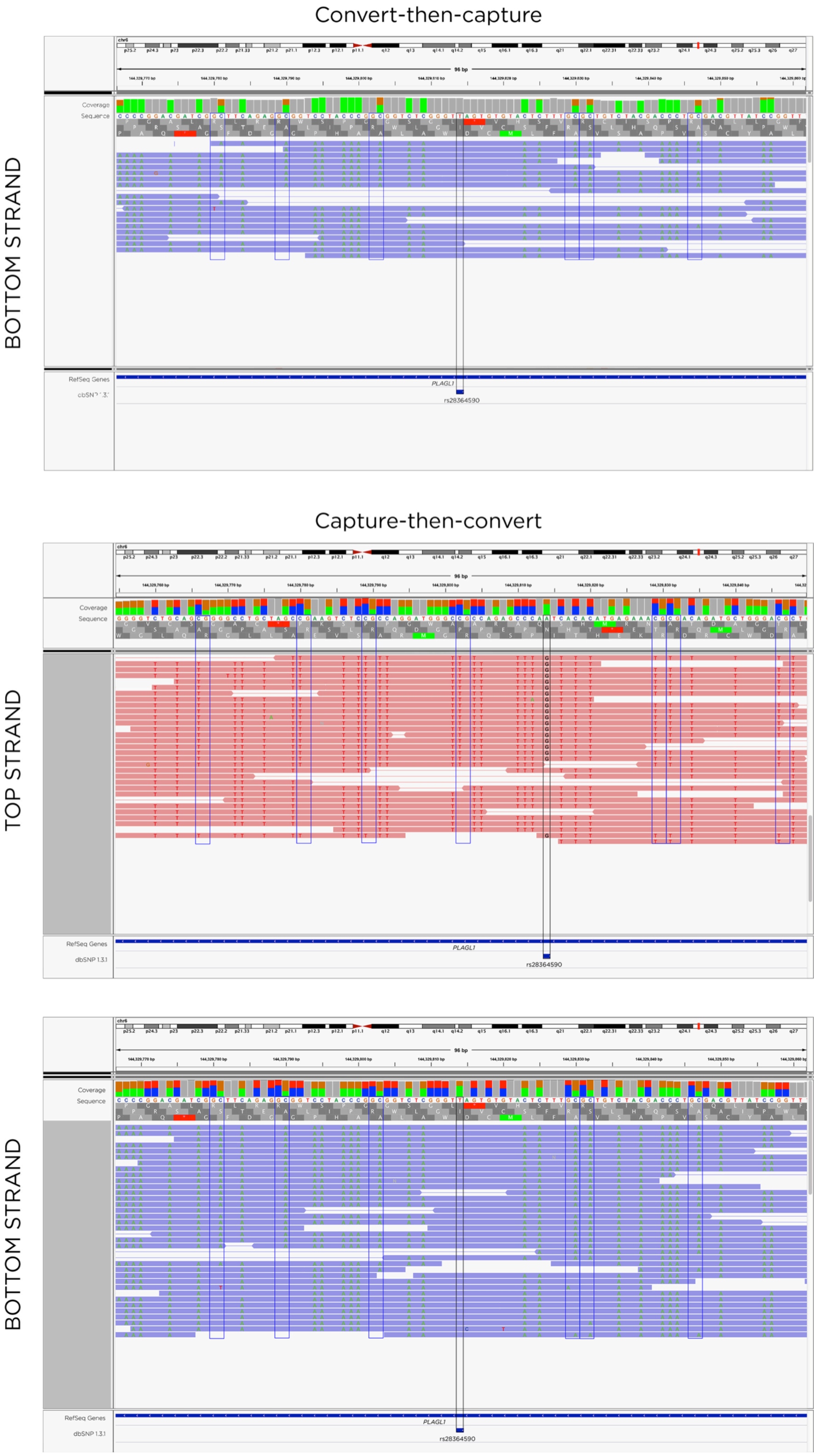

SUPPLEMENTARY FIGURE S8: An A:G SNP (rs28364590, black box) at the *HYMAI* imprinted differentially-methylated region can be detected in bisulphite reads from the top strand (minor G allele shown in black) but not the bottom, as the complementary C and T are indistinguishable following bisulphite treatment. The commercial capture-then-convert kit only captures one strand and consequently fails to recognize allelic DNA methylation at the subset of loci exemplified by the one shown here.
